# Cross-Cultural Adaptation and Validation of the Dundee Polyprofessionalism Inventory I: Academic Integrity among Brazilian Medical Students

**DOI:** 10.1007/s40670-026-02710-x

**Published:** 2026-03-26

**Authors:** Ahmed Haydar, Samira Yarak, Luwa Haidar, Gabrielle Leite Silveira, Paula Poco, Maria Teresa Riggio de Lima Landman, Lucas Albuquerque Chinelatto, Vinicius Gaby Vieira Rego, Milton de Arruda Martins

**Affiliations:** 1 Department of Neurology, University of São Paulo, São Paulo, Brazil; 2 Department of Dermatology, Federal University of São Paulo, São Paulo, Brazil; 3 American University of Beirut Faculty of Medicine (AUBFM), Beirut, Lebanon; 4 São Leopoldo Mandic School of Medicine, Campinas, Brazil; 5 Department of Pharmacology, Federal University of São Paulo, São Paulo, Brazil; 6 Department of Otorhinolaryngology, University of São Paulo, São Paulo, Brazil; 7 Department of Psychiatry, University of São Paulo, São Paulo, Brazil; 8 Center of Development of Medical Education, Department of Internal Medicine, University of São Paulo, São Paulo, Brazil

**Keywords:** Medical education, Professionalism, Cross-cultural comparison, Psychometric

## Abstract

**Introduction:**

The Dundee Polyprofessionalism Inventory I: Academic Integrity is a formative, non-scaling, descriptive test used to promote self-assessment, reflection and to evaluate students’ perception on academic misconduct. We report the framework and multistage methodological process of cross-cultural adaptation to Brazilian Portuguese and present validity evidence linking the instrument to educational use.

**Method:**

Following an 11-stage adaptation and validation process, we gathered: (i) content evidence via a 13-member expert panel using Lawshe’s Content Validity Ratio (CVR); (ii) qualitative response-process evidence via cognitive debriefing with 65 students; and (iii) reliability estimates from an online administration to 582 medical students (years 1–6) from public and private schools nationwide. Internal consistency was estimated with Cronbach’s α, and McDonald’s ω.

**Results:**

After iterative revision, all items met the critical CVR for 13 judges (≥ 0.538, *p* < 0.05). Cognitive debriefing improved role framing and scenario specificity. Reliability was high (α = 0.921; ω = 0.924). Students clearly understood the ten-level sanction hierarchy and considered the scenarios credible and relevant to the academic environment.

**Conclusion:**

The Brazilian version of Dundee Polyprofessionalism Inventory I: Academic Integrity shows strong content and response-process evidence and high reliability estimates, supporting its use. Its formative, scenario‑based design provides a culturally attuned tool for teaching and longitudinally tracking perceptions, prompting reflection and guiding educational decisions.

**Supplementary Information:**

The online version contains supplementary material available at 10.1007/s40670-026-02710-x.

## Introduction

Professionalism is a cornerstone in medical education due to its importance in patient satisfaction by increasing trust, reducing discomfort and increasing symptom resolution [1–6]. There has been growing interest in understanding how to teach and assess professional competence in health professions education [7–9]. Nevertheless, professionalism is a complex and unmeasurable concept, assessed indirectly through interpretation of observable behaviors and attitudes [10]. As a result, educators have increasingly incorporated psychometric principles into the evaluation of professionalism to improve the quality of measurement and interpretation of student behavior [11, 12].

Psychological tests offer a structured approach to capturing such behaviors and comparing responses among individuals. Despite their usefulness, most instruments are developed for English-speaking populations and may not account for diverse sociocultural contexts [13, 14].

To ensure valid application across different cultural settings, tools assessing professional behavior must undergo a rigorous process of cross-cultural adaptation. Although the Dundee Polyprofessionalism Inventory I: Academic Integrity has been applied in several cultural settings, no publication to date has described the full cross-cultural adaptation and validation methodology, limiting its replicability and cultural sensitivity in non-English contexts.

To address this gap, we conducted a multistage cross-cultural adaptation and validation of the Inventory for Brazilian medical students. This represents a unique contribution by providing the first comprehensive methodological description for a Portuguese-speaking population, tailored to Brazil’s diverse sociocultural and educational landscape.

**Fig. 1:**
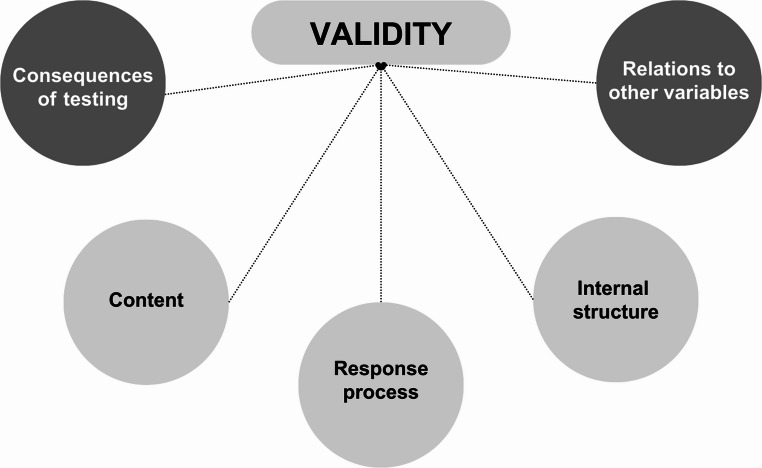
Sources of validity evidence assessed in this study. The evaluation focused on content, response process, and internal structure; evidence regarding relations to other variables and consequences of testing was not addressed

This article presents the methodological framework and stepwise process followed to collect evidence on content (linguistic and conceptual equivalence), response-process and structural validity (internal structure and consistency) of the adapted version [15, 16] (Fig. 1).

To achieve this, we addressed the following research questions in a step-by-step manner:

1. Content validity: How can a translated version ensure linguistic equivalence to the original version?
2. Content validity: Is there significant content validity of the adapted version in comparison to the original version, evaluated by a committee of experts using Lawshe’s Content Validity Ratio (CVR)?
3. Response process validity: How does qualitative feedback from pilot tests and cognitive debriefing with students refine the instrument for the Brazilian context?
4. Internal structure validity: In a larger sample, what is the internal consistency of the final version, measured by Cronbach’s α and McDonald’s ω, and internal structure measured by confirmatory factor analysis (CFA)?

This sequential approach ensures that the instrument maintains its theoretical integrity while adapting to the Brazilian sociocultural context. 

## Theoretical Framework: Protoprofessionalism

Because professionalism cannot be measured directly, its evaluation relies on measurable indicators, proxies. A theoretical framework connects these indicators to the concept of professionalism [12, 15]. However, there is currently no consensus on a single unifying theory [16–18].

Therefore, we adopted Hilton and Slotnick’s view, which defines professionalism as “a state reached only after a prolonged period of learning, instruction and reflective experience”. This developmental phase where students acquire knowledge, skills and reflective experience to achieve maturity is described as “proto-professionalism” [15, 16]. In this framework, professionalism is seen as practical wisdom, situated within the attitudes domain of Bloom’s taxonomy, developing through experience and reflection, which emphasizes metacognition and reflective thinking as essential to professional identity formation during “proto-professionalism” [17, 18].

## Ethical Approval

In compliance with the World Medical Association Declaration of Helsinki this study was approved by the Faculty of Medicine of the University of São Paulo’s ethical committee (protocol number CAAE: 50517421.3.0000.0068). Those who agreed to participate signed a free, prior, and informed consent form after hearing an explanation of the study’s objectives.

## Methods: Cultural Adaptation and Validation Steps

This cross-sectional, observational, descriptive-analytic study utilized a questionnaire-based approach to adapt and validate the 34-item version of the Dundee Polyprofessionalism Inventory I: Academic Integrity for Brazilian medical students [19–25].

**Fig. 2:**
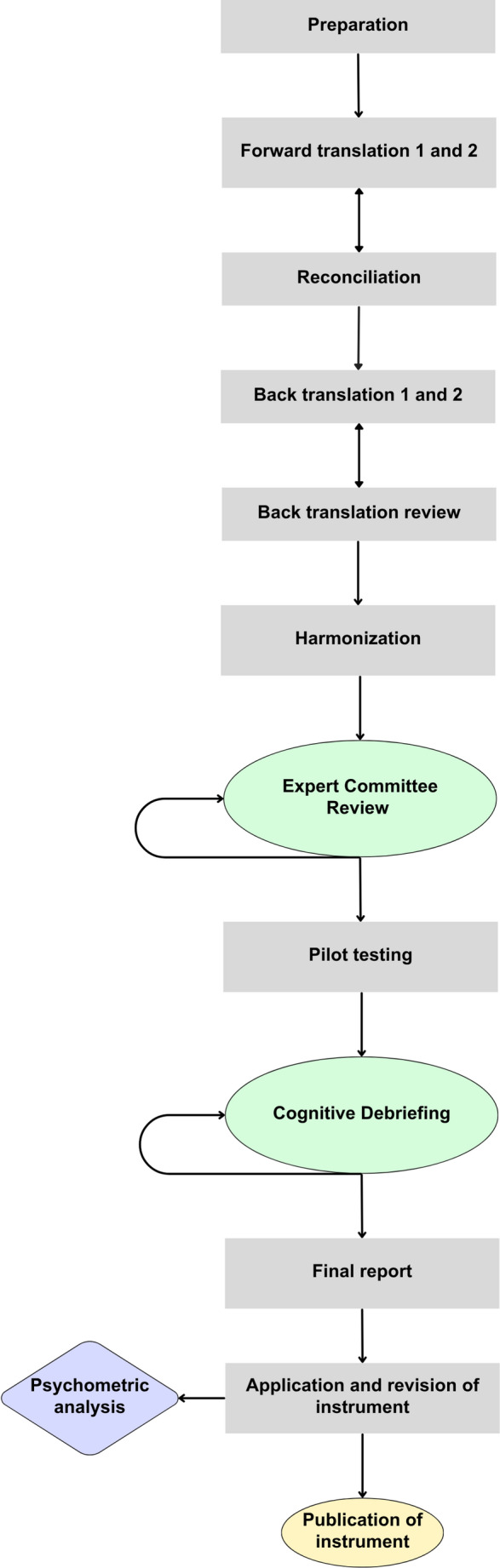
Overview of the multistage methodological framework used for adaptation and validation. (adapted from Beaton and Patient-Reported Outcome Measurement Information System (PROMIS^®^) scientific standards) [27]

The respondents received 34 unprofessional scenarios and were asked to recommend appropriate sanctions for a first time infraction with no mitigating circumstances from a hierarchy of ten options, which are progressive in severity and not necessarily aligned with institutional regulations [26]. Response data was collected via an online platform (Google Forms^®^) between 2021 and 2024. To address the research questions outlined in the Introduction, this section details the multistage process applied to ensure linguistic, cultural, and psychometric validity [13, 14]. This article followed the sequential approach proposed by Beaton and by the Patient-Reported Outcome Measurement Information System (PROMIS^®^) scientific standards [13, 27] (Fig. 2). All steps respected the principles of good practice proposed by the Professional Society for Health Economics and Outcomes Research (ISPOR) Task Force [28]. A detailed table is available in Supplemental Material 1 and can be used as a blueprint.

### Steps 1–6: Linguistic Equivalence (Addressing Research Question 1)

#### 1. Preparation

Authorization was obtained from the inventory’s developer. A Project Manager (AH) and a Committee of Consultants (SY, MT, LC, VG) and Proofreaders (MT) were appointed.

#### 2. Forward Translation

Two independent professional translators, native speakers of the target language, translated the inventory from English to Brazilian Portuguese (FT1 and FT2).

#### 3. Reconciliation

The Project Manager, with the Committee of Consultants and the Forward Translators reconciled the translations (FT1.2).

#### 4. Back Translation

Two independent professional translators, native English speakers, translated the reconciled version (FT12) from Brazilian Portuguese to English (BT 1 and BT2).

#### 5. Back Translation Review

The Project Manager, with the Committee of Consultants reviewed and reconciled the back translations into a single version (BT1.2).

#### 6. Harmonization

All translations were compared with the original inventory, with minor adjustments made in consultation with the developer to ensure equivalence.

### Step 7: Content Validity (Addressing Research Question 2)

#### 7. Expert Committee Review

A 13-member Brazilian Portuguese-speaking panel (health professionals, medical educators, linguist, translator) was assembled to assess Content Validity [11, 29]. They judged four linguistic domains (semantic, idiomatic, experiential, conceptual) and three additional domains (clarity, theoretical relevance, practical pertinence). Lawshe’s Content Validity Ratio (CVR) was used to quantify agreement [30, 31]. The minimum agreement rate to surpass the chance of type I error (α of 0.05), known as critical CVR, is 0.538 considering the number of judges in the Committee (critical CVR = 0.538, α = 0.05).

#### Steps 8–10: Response Process Validity (Addressing Research Question 3)

#### 8. Pilot Testing

The Inventory was initially applied to 65 students from two different public universities to verify if the adapted version preserved its equivalences in a real-world scenario [13].

#### 9. Cognitive Debriefing

Two cognitive debriefing meetings were held immediately after the pilot testing sessions, with a total of 65 students participating, to explore the cognitive rationale behind their responses to the inventory [27].

#### 10. Final Report

A final report describing the process with the final version was sent to the Developer to allow a process audit [13].

### Step 11: Internal Structure Validity (Addressing Research Question 4)

#### 11. Application and Psychometric analysis (Addressing Research Question 4)

The final version of the Inventory was applied to a non-probabilistic convenience sample of 582 Brazilian medical students, from first to sixth year, enrolled in public and private medical schools across different regions of the country. Recruitment occurred via online invitations to participating institutions, reflecting Brazil’s sociocultural and educational diversity, including its community-oriented medical curriculum, to ensure relevance to local ethical expectations. To gather evidence for the inventory’s internal structure we followed the Standards for Educational and Psychological Testing [29]. Internal consistency was evaluated on the polychoric correlation matrix with two complementary coefficients: Cronbach’s α and McDonald’s total ω. Confirmatory (CFA), but not exploratory factor analysis (EFA) was planned [32–34]. All psychometric analyses were run in JAMOVI v 2.3.28.

### Qualitative Evidence Collection and Statistical Analysis

**Fig. 3:**
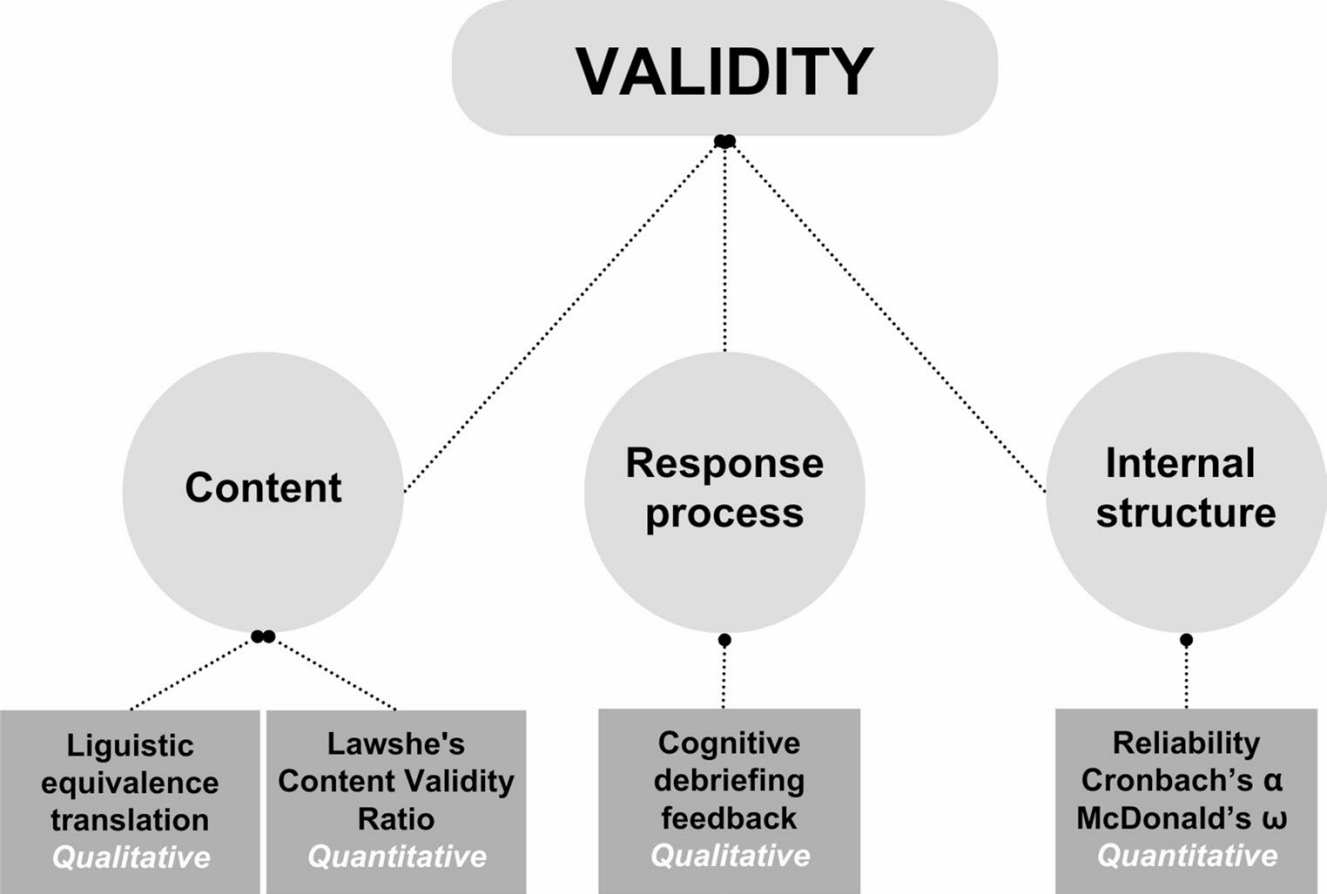
Summary of data sources supporting each validity domain assessed in this study

Different data sources of validity evidence were analyzed (Fig. 3). Steps 1–6 (translation, reconciliation, back-translation, review, and harmonization) produced the initial Brazilian Portuguese version. Step 7 generated statistical evidence of content validity via Lawshe’s CVR from a 13-member expert panel. Step 9 gathered qualitative response process evidence through cognitive debriefing. Step 11 provided statistical reliability estimates (Cronbach’s α and McDonald’s ω) from the full administration, with confirmatory factor analyses pre-specified for assessment of internal structure.

## Results

### Expert Committee Review (Aligned with Research Questions 1 and 2)

Inventory instructions on “how to answer” and three items were below critical CVR in the linguistic equivalence domain (data with CVR available in Supplemental Material 2). Revised items were redjudged and surpassed the threshold. The panel agreed on the inventory’s population scope: students and teachers of healthcare professionals, but not for healthcare professionals compliance assessment. No agreement on dimensionality was reached.

### Cognitive Debriefing Qualitative Data Data to Refine the Instrument Based on Feedback (Aligned with Research Question 3)

The main modifications suggested by the students were aimed at enhancing the clarity and understandability of the inventory.

Students requested clearer role specification and scenario specificity. Initially, the instructions on “how to answer” the inventory were considered difficult to understand, and clearer guidance was needed (reported by 23/65 participants). Additionally, students asked for more details regarding their role in the academic institution, whether they should assess the actions from the perspective of a student, a director, or a teacher. This explanation was required to guarantee that responders could appropriately judge the seriousness of the acts being detailed.

For instance, in response to the scenarios about “drugs & violence,” participants expressed the need to specify that the use is happening in the academic environment (reported by 5/65 participants). Similarly, in the section about alcohol consumption, students requested clarification on the level of alcohol intake, specifically if the person was intoxicated or not during patient encounters (reported by 14/65 participants).

This feedback was used to revise the inventory to make sure every scenario was precise and unambiguous, increasing the accuracy and reliability of the responses. All modifications were reviewed by the developer to preserve intended meanings.

### Application and Psychometric Analysis to Evaluate the Internal Structure and Consistency of the Final Version (Aligned with Research Question 4)

The Inventory was administered to 582 Brazilian medical students (Table 1 for demographic details). This process enabled the analysis of the internal structure. The psychometric item analysis data indicate dimensionality, reliability, and factor analysis. Data showed good reliability (Cronbach’s α = 0.921 and McDonald’s ω = 0.924). Item analysis showed that propositions exclusion of items 4, 5, 7, 15, 23 and 28 would marginally increase Cronbach’s α and won’t change McDonald’s ω.

**Table 1:** Demographic data of non-probabilistic convenience sample of Brazilian medical students

Variable	Statistics
Age (years)	Median 20 years (IQR 19–22)
Gender	Female 69% (402/582); Male 31% (180/582)
Ethnicity	White 78% (456/582); Brown/Black 18% (105/582); Yellow 3% (21/582)
Year of School	1st and 2nd years: 22% (131/582); 3rd and 4th years: 66% (383/582); 5th and 6th years: 12% (68/582)
Region of Medical School	Urban 52% (307/582); Rural 48% (275/582)
Type of institution	Private: 87% (507/582); public: 13% (75/582)

We ran a sensitivity, unidimensional CFA and found inadequate fit to justify a common-factor scale: Chi-square = 2495 (df = 527, *p* < 0,001); RMSEA = 0.0802 (0.077–0.0833); CFI = 0.705; TLI = 0.686; SRMR = 0.0696. 

## Discussion

This study is the first to report the full cross-cultural adaptation and psychometric evaluation of the Dundee Polyprofessionalism Inventory I: Academic Integrity for Portuguese-speaking medical students in Brazil. Given that perceptions of academic misconduct vary across cultures, a direct translation of the original instrument would risk compromising diagnostic precision and pedagogical relevance [13]. Cross-cultural application in non-English speaking countries allowed the identification of different patterns in students from Saudi Arabia, Egypt, Pakistan and United Arab Emirates, compared to European students [19, 21, 23–25, 35–39]. In Brazil, where sociocultural diversity and unique medical education structures shape ethical expectations, assessing professionalism requires culturally tailored tools.

Qualitative data gathered during Expert Review and Cognitive Debriefing confirmed that Brazilian students clearly understood the ten-level sanction hierarchy and considered the scenarios credible and relevant to the academic environment.

Following a rigorous 11-stage process, the Brazilian version demonstrated strong content validity (all CVR values ≥ 0.538 after iterative revision) and excellent internal consistency (α = 0.921; ω = 0.924), supporting its reliability in this context.

A key consideration is the interpretation of construct validity findings. Due to the formative nature of the instrument, items assess distinct facets of academic integrity rather than reflecting a single, underlying latent trait. Consistent with this design, the expert panel did not endorse a unified reflective dimension, and prior applications of the inventory have not established dimensional structure (16,19–25,35–42). Consequently, a total score is not calculated; instead, each item provides specific, actionable information. In factor analysis, the unidimensional CFA model resulted in poor fit indices, not allowing to treat the inventory as a common-factor scale. We did not interpret the CFA further, avoiding imposition of an ill-fitting reflective model [32, 33].

This result may be attributed to the nature of the Dundee scale. It is designed as a formative rather than a reflective scale, therefore, standard structural analysis does not apply. Items in reflective scales represent the evaluated domain, meaning the construct remains stable if an item is removed. In contrast, items in a formative scale determine specific aspects of the construct. Therefore, item exclusion changes the measurement’s interpretation. Consequently, a total score is not calculated; instead, each item provides specific information [40].

For instance, a factor that groups “*Cheating in an exam by e.g. copying from neighbour*,* taking in crib material or using mobile phone or getting someone else to sit for you*” with “*Lack of punctuality for classes*” may emerge statistically due to spurious correlations, but it does not represent a coherent educational construct, as these behaviors stem from distinct facets of professionalism (e.g., integrity vs. punctuality). Additionally, while items such as “*Claiming collaborative work (group work) as one’s individual effort*” and “*Completing work for another student*” sound similar and might even be considered redundant candidates for removal to enhance internal validity, they assess different aspects of plagiarism and are not interchangeable.

Given these results, pursuing further dimensional analyses, such as EFA, would violate core psychometric assumptions and risk producing statistically artificial and educationally uninterpretable factors [34].

The importance of our findings is not based solely on internal structure evidence but is instead robustly supported by other essential forms of validity. These include content validity, established through expert judgment on the relevance and linguistic equivalence of the items; response-process validity, confirmed via cognitive debriefing with participants; and response coherence by the reliability indices result. This multifaceted approach ensures that our conclusions are grounded in a psychometrically sound application of the inventory.

Unlike most self-administered professionalism instruments that employ a scaling and classificatory approach, the Dundee Poly Professionalism Inventory I: Academic Integrity adopts a diagnostic and descriptive format [16, 41]. Rather than assigning a score or a category, the inventory allows educators and students to explore perceptions of academic lapses in a formative and reflective manner. In Brazilian medical schools, the adapted version offers a practical, ready-to-use formative tool that can support guided discussions, curricular integration, and ethics-focused feedback sessions [42, 43]. Importantly, the inventory also enables longitudinal use across the medical curriculum. By administering it at multiple points during medical school, educators can monitor changes in students’ perceptions of academic integrity and professionalism. This supports the development of professional identity over time, aligning with competency-based frameworks and fostering self-awareness in the proto-professional phase [24].

The relevance of formative and culturally adapted tools for the Brazilian context is corroborated by previous research. For instance, the Professional Identity Essay (PIE), an open-ended narrative instrument, was recently translated, adapted, and validated for use in Brazil [44]. The PIE’s validation process followed a similar multistage methodology, including content validation and the decision to forego factor analysis due to the formative and non-classificatory nature of the instrument. In that study, the PIE was well received by students and demonstrated the potential to stimulate longitudinal reflection and professional growth when used in feedback-guided sessions. Similarly, the Dundee Polyprofessionalism Inventory I offers educators a structured, theory-informed method to encourage ethical awareness and critical thinking. Both instruments contribute to a growing repertoire of culturally sensitive strategies that support the development of professionalism in diverse sociocultural contexts.

To substantiate validity based on relations to other variables, future studies could analyze the convergence between this inventory and qualitative measures, such as the Professionalism Identity Essay. This would allow researchers to triangulate quantitative scores with categorical developmental levels of professional identity.

Furthermore, regarding validity based on consequences of testing, longitudinal studies are needed to assess distinct cohorts over time. Researchers should investigate whether implementation correlates with a reduction in objective academic integrity incidents, such as plagiarism rates or exam cheating reports, compared to control groups. Finally, the instrument’s predictive power could be evaluated by linking student performance to long-term professional outcomes, specifically by comparing test results against historical data on disciplinary events and complaints lodged with medical boards.

## Limitations

Several versions of the Inventory have been published. The absence of a standardized number of items limits comparisons across cohorts. The original 41-item form [16,41,45, 46], a 30-item subset used in some studies [35, 37, 38], and the now more common 34-item version [20–25, 39] are available. We selected the 34-item version after critically reviewing the items removed from the original scale. Extensive discussions indicated that deleting certain items did not compromise the instrument’s validity while allowing shorter responder time; however, the resulting 30-item would be overly concise and possess reduced power. Furthermore, the 34-item version is better established and has been consistently employed in the literature, making it the most suitable choice for our study and for future cohort comparisons.

Finally, although the sample did reflect broad regional and institutional diversity, the use of a non-probabilistic sample constrains the generalizability of findings. A more rigorous design would have involved random selection of schools followed by sampling of students within. Implementing such a design was impracticable because data collection was conducted entirely online and participation of students was voluntary. These constraints introduce potential self-selection bias, unevenly representing public versus private schools, students from different years of medical school or even social backgrounds, reducing the sample’s representativeness across these important educational variables.

## Conclusion

The Dundee Poly Professionalism Inventory I: Academic Integrity is a non-scaling and non-classificatory psychological instrument that enables a descriptive analysis of professionalism and insight into different students’ perceptions on academic lapses. Moreover, longitudinally, students can self assess themselves and reflect during their proto-professional developmental phase. Cross-cultural adaptation and validation of the Inventory, following a rigorous multistage process is essential to ensure applicability and preserve psychometric properties across cultures.

With this validated version, Brazilian medical schools can now incorporate the Inventory into ethical discussions or professionalism workshops to guide conversations about academic lapses. Longitudinally, these discussions can raise attention if students are aligned with local social expectations on how a healthcare professional is. It can as well allow comparison with educators’ patterns of responses and even understanding of generational differences in behaviors.

Future research could explore whether changes in students’ recommended sanctions over time correlate with behaviors in clinical settings. Additionally, studies employing random sampling of schools, and subsequently students within those schools, could enhance the representativeness and generalizability of findings. 

## Supplementary Information

Below is the link to the electronic supplementary material.

Supplementary Material 1 (XLSX 15.5 KB)

Supplementary Material 2 (PDF 67.3 KB)

## Data Availability

The datasets used and/or analyzed during the current study are available from the corresponding author on reasonable request.
