# Supplementary material for: Cross-Cultural Adaptation and Validation of the Dundee Polyprofessionalism Inventory I: Academic Integrity among Brazilian Medical Students
Source: Med Sci Educ. 2026 Mar 26;36(3):1489–98. doi: 10.1007/s40670-026-02710-x (PMC13356110; doi:10.1007/s40670-026-02710-x)
Supplement: Supplementary file 2 — Supplementary Material 2 (PDF 67.3 KB) [file 40670_2026_2710_MOESM2_ESM.pdf]

ORIGINAL INGLÊS	BRAZILIAN PORTUGUESE DRAFT	Linguistic equivelence (sum of domains - Semantic, Idiomatic, Experiential and Cultural)	Clarity	Theoretical relevance	Practical pertinence
Recommend appropriate sanctions for a first time infraction with no mitigating circumstances from the following hierarchy of options, which are theoretical rather than necessarily available within the regulations in each of the medical schools	Recomende sanções adequadas a um primeiro infrator sem circunstâncias atenuantes a partir da seguinte hierarquia de opções, que são teóricas e não necessariamente disponíveis nos regulamentos de cada um dos cursos da saúde	0,3333333333	0,3333333333	1	1
1 Ignore	1 Ignorar	1	1	1	1
2 Reprimand (verbal warning)	2 Advertir verbalmente	1	1	1	1
3 Reprimand (written warning)	3 Advertir por escrito	1	1	1	1
4 Reprimand, plus mandatory counselling	4 Advertir e exigir adesão à programa de orientação psicopedagógica	0,6666666667	1	1	1
5 Reprimand, counselling, extra work assignment	5 Advertir, exigir adesão à programa de orientação psicopedagógica e atribuir trabalho adicional	0,6666666667	1	1	1
6 Failure of specific class/remedial work to gain credit	6 Reprovar em matéria específica ou exigir trabalho de recuperação para aprovação	1	1	1	1
7 Failure of specific year (repetition allowed)	7 Reprovar em ano específico com permissão para refazer o ano	1	1	1	1
8 Expulsion from college (readmission after one year possible)	8 Expulsar do curso com possibilidade de readmissão após um ano	1	1	1	1
9 Expulsion from college (no chance for readmission)	9 Expulsar do curso sem possibilidade de readmissão	1	1	1	1
10 Report to regulatory body	10 Denunciar ao conselho de classe para proibição do exercício profissional	0	0,6666666667	1	0,8181818182
Getting or giving help for course work, against a teacher's rules (e.g. Lending work to another student to look at)	Receber ou oferecer ajuda a outro estudante para qualquer trabalho do curso, contrariando as regras do professor (por exemplo, emprestando trabalho para que outro estudante veja)	0,8333333333	1	1	1
Removing an assigned reference from a shelf in the library in order to prevent other students from gaining access to the information in it	Retirar da estante da biblioteca livros ou referências indicados ou barrar acesso à biblioteca digital para dificultar o acesso de outros estudantes às informações neles contidas	0,6666666667	0,8333333333	0,8181818182	0,8181818182
Signing attendance sheets for absent friends, or asking classmates to sign attendance sheets for you in labs or lectures	Assinar folhas de presença para estudantes ausentes ou pedir a outros estudantes que assinem folhas de presença em laboratórios, aulas ou qualquer atividade didática	0,1666666667	0,6666666667	1	1
Drinking alcohol over lunch and interviewing a patient in the afternoon	Atender ou entrevistar paciente após a ingestão de bebida alcoólica	0,6666666667	1	1	1
Exchanging information about an exam before it has been taken (e.g. OSCE)	Passar ou pedir informações sobre uma avaliação antes que esta tenha sido realizada (por exemplo, Exame Clínico Objetivo Estruturado - OSCE)	1	1	1	1
Forging (copying) a healthcare worker's signature on a piece of work, patient chart, grade sheet or attendance form	Forjar (copiar) a assinatura de um profissional de saúde ou professor num trabalho, prontuário de paciente, folha de avaliação ou formulário de presença	0,8333333333	1	1	1
Claiming collaborative work (group work) as one's individual effort	Reivindicar trabalho em grupo (colaborativo) como um esforço individual	0,6666666667	0,6666666667	1	1
Altering or manipulating data (e.g. adjusting data to obtain a significant result)	Alterar ou manipular dados (por exemplo, ajustar dados para obter um resultado significativo)	1	1	1	1
Failure to follow proper infection control procedures	Falhar em seguir procedimentos adequados de controle de infecção	1	1	1	1
Threatening or verbally abusing a university employee or fellow student	Ameaçar ou assediar verbalmente de um funcionário da universidade ou outro estudante	0	0,8333333333	1	1
Attempting to use personal relationships, bribes (illegal rewards) or threats to gain academic advantages by getting advance copies of exam papers or passing an exam by such pressures on staff	Tentar usar contatos pessoais, subornos (recompensas ilegais) ou ameaças para obter vantagens acadêmicas por meio da obtenção prévia de cópias das avaliações ou aprovação em avaliações por pressões sobre funcionários	1	0,8333333333	1	1
Engaging in substance misuse (e.g., drugs)	Fazer uso indevido de substâncias ilícitas (por exemplo, drogas)	0,6666666667	0,8333333333	0,8181818182	0,8181818182
Completing work for another student	Realizar um trabalho por outro estudante	1	1	1	1
Intentionally falsifying (changing) test results or treatment records in order to disguise (hide) mistakes	Falsificar (alterar) resultados de testes ou prontuários a fim de disfarçar (ocultar) erros	1	1	1	1
Physically assaulting a university employee or student	Agredir fisicamente um funcionário ou estudante da universidade	1	1	1	1
Purchasing (buying) work from a fellow student or Internet etc. supplier	Adquirir (comprar) trabalho de um outro estudante ou fornecedor da Internet etc.	1	1	1	1
Lack of punctuality for classes	Falta de pontualidade para as aulas	1	1	0,8181818182	0,8181818182
Providing illegal drugs to fellow students	Fornecer substâncias ilícitas a outros estudantes	1	1	1	1
Not doing the part assigned in group work	Não realizar a parte que lhe foi atribuída num trabalho de grupo	1	1	1	0,8181818182
Examining patients without knowledge or consent of supervising clinician	Examinar pacientes sem conhecimento ou consentimento do profissional de saúde supervisor	1	1	1	1

ORIGINAL INGLÊS	BRAZILIAN PORTUGUESE DRAFT	Linguistic equivelence (sum of domains - Semantic, Idiomatic, Experiential and Cultural)	Clarity	Theoretical relevance	Practical pertinence
Sabotaging (deliberately damaging) another student's work	Sabotar (danificar deliberadamente) o trabalho de outro estudante	1	1	1	1
Inventing extraneous (irrelevant) circumstances to delay sitting an exam	Inventar circunstâncias alheias para atrasar a realização de uma avaliação	0,8333333333	0,8333333333	1	1
Sexually harassing a university employee or fellow student	Assediar sexualmente um funcionário ou outro estudante	0,8333333333	1	1	1
Resubmitting work previously submitted for a separate assignment or earlier degree	Entregar um trabalho que já tenha sido previamente entregue em uma outra tarefa ou em um outro curso	1	1	1	0,8181818182
Plagiarising work (stealing ideas and presenting them as one's own) from a fellow student or publications/Internet	Plagiar trabalho (roubar ideias e apresentá-las como próprias) de outro estudante ou de publicações ou Internet	1	1	1	1
Cheating in an exam by e.g. copying from neighbour, taking in crib material or using mobile phone or getting someone else to sit for you	Colar numa avaliação, como por exemplo copiar de estudante ao lado, trazer anotações, usar o telefone celular ou pedir a outra pessoa para realizar a sua avaliação	0,8333333333	0,8333333333	1	0,8181818182
Cutting and pasting or paraphrasing material without acknowledging the source	Copiar e colar ou parafrasear material sem citar a fonte	0,8333333333	1	1	1
Damaging public property, e.g., scribbling on desks or chairs	Danificar propriedade de uso comum, por exemplo, rabiscar carteiras ou cadeiras	1	1	1	1
Falsifying personal references (recommendation letters) or grades on a curriculum vitae or altering grades in the official record	Falsificar referências pessoais (cartas de recomendação), notas num curriculum vitae ou alterar notas no registro oficial	0,8333333333	1	1	0,8181818182
Involvement in paedophilic activities – possession/viewing of child pornography images or molesting children	Envolver-se em atos de pedofilia - como possuir ou visualizar imagens de pornografia infantil ou abusar de crianças	1	1	1	1
Photographing dissection or pro-section or cadaver materials	Fotografar materiais de dissecação, prossecção ou cadáveres	1	1	1	1
Joking or speaking disrespectfully about bodies/body parts	Ridicularizar ou falar desrespeitosamente de corpos ou partes do corpo.	0,6666666667	0,6666666667	1	1
Inappropriate representation of Medicine in social media by posting photos/videos/texts about class or clinic activities	Representar inadequadamente as profissões da saúde nas redes sociais postando fotos, vídeos ou textos sobre aulas ou atividades clínicas	1	1	1	1
Posting inappropriate material about fellow students, teachers or patients on social media	Postar material impróprio sobre estudantes, funcionários ou pacientes nas redes sociais	1	1	1	1
